# DCTPP1 orchestrates dCTP pool dynamics and mtDNA stability in quiescent cells

**DOI:** 10.1038/s41419-026-08632-1

**Published:** 2026-03-26

**Authors:** Belén Fernández, Guiomar Pérez-Moreno, Blanca Martínez-Arribas, Antonio E. Vidal, Luis Miguel Ruiz-Pérez, Dolores González-Pacanowska

**Affiliations:** Instituto de Parasitología y Biomedicina “López-Neyra” (IPBLN), CSIC, Parque Tecnológico de Ciencias de la Salud. Avda. del Conocimiento, Granada, Spain

**Keywords:** DNA metabolism, Mechanisms of disease

## Abstract

Defects in nucleotide metabolism and imbalances in deoxynucleotide triphosphate (dNTP) pools are associated with several human diseases, including cancer and mitochondrial disorders. In non-replicative cells, while DNA synthesis is reduced, a continuous supply of nucleotides is essential to sustain mitochondrial DNA (mtDNA) replication and repair. Human all-α dCTP pyrophosphatase 1 (DCTPP1), a nucleotido hydrolase with high specificity for dCTP, plays a critical role in maintaining nucleotide homeostasis, however its participation in mtDNA stability remains unexplored. In this study we performed a detailed analysis of pyrimidine metabolism enzymes in non-dividing cells. We found that during quiescence, DCTPP1 is predominantly localized to mitochondria. Depletion of the enzyme leads to upregulation of the de novo thymidylate synthesis pathway and expansion of both the dCTP and dGTP pools, highlighting its pivotal role in regulating the dNTP balance. To explore the potential therapeutic relevance of these observations, we used an in vitro model of mitochondrial neurogastrointestinal encephalomyopathy (MNGIE), a rare mitochondrial disorder caused by thymidine phosphorylase (TP) deficiency and characterized by dCTP depletion and mtDNA loss. Long-term thymidine overloading in quiescent cells (a model mimicking TP deficiency) led to reduced dCTP levels and the depletion of mtDNA, effects that were reversed upon DCTPP1 knockdown. Hence, reduced DCTPP1 levels restored dCTP availability and increased mtDNA copy number. These findings suggest that DCTPP1 plays a critical role in regulating mitochondrial dNTP pools and that down-regulation of the enzyme may serve as a compensatory mechanism in disorders marked by secondary dCTP depletion. DCTPP1 may therefore represent a promising therapeutic target for mitochondrial DNA depletion syndromes such as MNGIE.

## Introduction

A balanced supply of dNTPs is essential for the accurate progression of DNA replication and repair within both the nuclear and mitochondrial compartments [1–3]. The size and composition of dNTP pools vary depending on the cellular state and are tightly regulated by coordinated anabolic and catabolic pathways [4]. In proliferating cells, dNTP synthesis is upregulated during the S phase of the cell cycle while in non-dividing cells, the demand for nucleotide precursors is markedly reduced, rendering dNTP levels largely independent of cell division. Outside the S phase or during quiescence, dNTPs are primarily required for mitochondrial DNA (mtDNA) replication and DNA repair [5]. Despite the overall reduction in dNTP levels in non-dividing cells, a finely tuned network of enzymes governing dNTP metabolism is crucial to maintain a steady supply for mtDNA replication and maintenance. Defects in genes associated with dNTP synthesis and mtDNA replication, or imbalances in mitochondrial dNTP pools, lead to mitochondrial DNA depletion syndromes (MDSs), a diverse group of disorders marked by mtDNA depletion and/or deletions in energy-demanding tissues such as muscle, liver, and brain [3, 6, 7].

In proliferating cells, mtDNA precursors are derived from both the salvage pathway and cytosolic de novo synthesis, whereas in quiescent cells, the salvage pathway predominates. A key enzyme in dNTP biosynthesis is ribonucleotide reductase (RNR), which catalyses the conversion of ribonucleoside diphosphates (rNDPs) to deoxyribonucleoside diphosphates (dNDPs), which are then phosphorylated to dNTPs by nucleoside diphosphate kinases. In mammals, RNR is a heterotetrameric complex composed of two subunits: the large catalytic R1 and the smaller regulatory R2 [8]. The R2 subunit is degraded during late mitosis [9], resulting in quiescent cells with low or undetectable R2 and residual levels of R1. In response to DNA damage, a homologue of R2, p53-induced ribonucleotide reductase subunit 2 (p53R2), is induced and performs the same catalytic function without undergoing cell-cycle-dependent degradation [10]. Within the salvage pathway, mitochondrial phosphorylation of deoxynucleosides represents a rate limiting step. Thymidine kinase 2 (TK2), which generates dCTP and dTTP, and deoxyguanosine kinase (DGUOK), which produces dGTP [3, 7] are essential for sustaining mitochondrial dNTPs in non-proliferative tissues.

In addition to synthesis and salvage, maintaining genetic integrity requires the removal of damaged or non-canonical nucleotides before their incorporation into DNA. This “house-cleaning” function is performed by a class of NTPases (nucleoside triphosphate pyrophosphatases) which hydrolyse damaged or non-canonical nucleoside triphosphates to their corresponding monophosphates. These enzymes fall into four structurally different superfamilies: nudix hydrolases, trimeric dUTPases (deoxyuridine triphosphate nucleotidohydrolases), ITPases (inosine triphosphate pyrophosphatases) and all-α NTP pyrophosphatases [11].

DCTPP1, an all-α NTP pyrophosphatase, hydrolyses 5’-modified dCTP derivatives yet has been shown to play a critical role in the hydrolysis of canonical dCTP [12]. Loss of DCTPP1 leads to dCTP accumulation, DNA damage and genomic instability [13]. The enzyme is also involved in the metabolism of deoxycytidine analogues used in cancer chemotherapy [14]. DCTPP1 is ubiquitously expressed, with higher levels in proliferative tissues, and is localized to the nucleus, cytosol, and mitochondria in dividing cells [12]. Its role in nucleotide metabolism has led to its consideration as a potential biomarker and therapeutic target in cancer [15]. However, its specific contribution to mitochondrial nucleotide homeostasis and mtDNA integrity remains poorly understood.

In this study, we investigate the potential role of DCTPP1 in regulating nucleotide levels and ensuring the fidelity of mtDNA replication. Using quiescent cells, where the de novo synthesis pathway is diminished and mtDNA replication and repair rely primarily on the salvage pathway, we analyse the impact of DCTPP1 depletion on dNTP pools. Additionally, we assess the expression of enzymes involved in dNTP metabolism to better understand the broader implications of DCTPP1 function. Our findings indicate that DCTPP1 is essential for maintaining the dNTP balance, and suggest that it may represent a therapeutic target for conditions such as MNGIE, a mitochondrial disorder linked to dNTP pool dysregulation.

## Results

### Pyrimidine metabolism in cycling versus quiescent CCD-34Lu cells

**Fig. 1:**
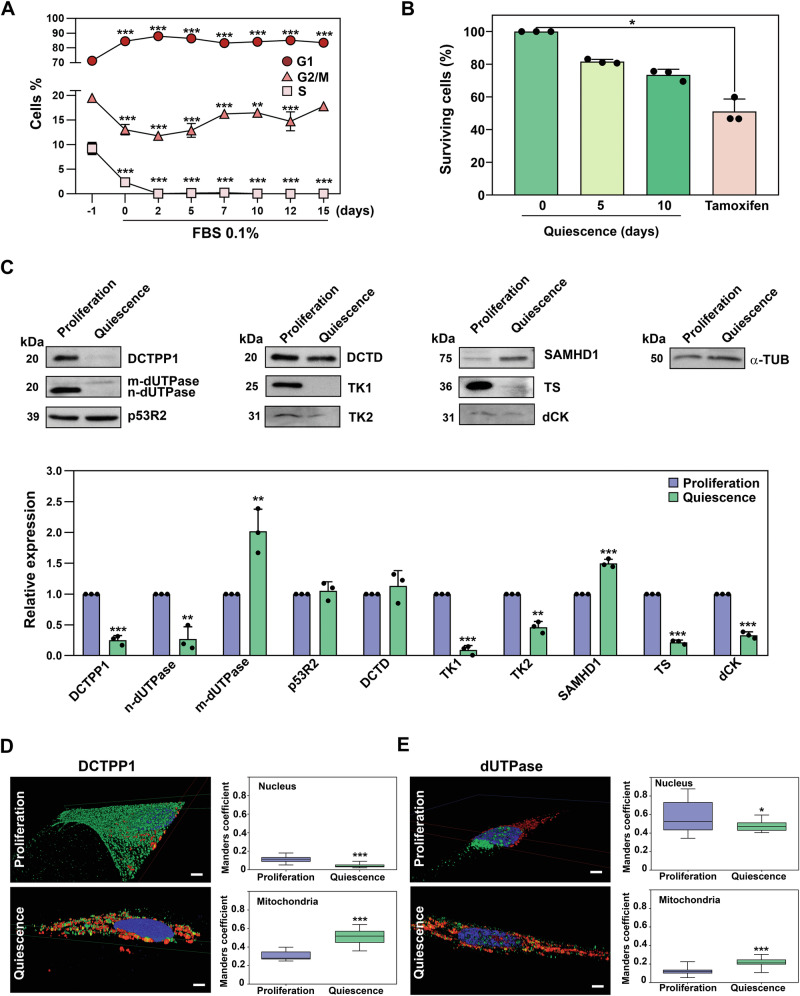
Pyrimidine metabolism in cycling versus quiescent CCD-34Lu cells. **A** Time-course cell cycle profiling of CCD-34Lu cells subjected to quiescence induction by serum starvation (0.1% FBS) over the indicated days (left panel). Data points represent the mean of three biological replicates (mean ± S.D.). Statistical analysis was performed using two-way ANOVA (F_(14, 30)_ = 100.80, *p* < 0.001), with Dunnett’s post hoc test for multiple comparisons. **B** Cell viability was determined using crystal violet live-cell staining in quiescent cells, expressed as the percentage of surviving attached cells. Tamoxifen (50 µM, 12 h) was used as a positive control for cell death. Results are shown as mean ± S.D. (*n* ≥ 3), with individual data points indicated. Kruskal Wallis test with Dunn’s multiple comparison correction was applied for the statistical analysis. **C** Western blot analysis of pyrimidine metabolism enzymes in resting versus proliferating cells (upper panel). Only relevant sections of the full-length blots are shown; original blots are available in the Supplementary File. Quantification (lower panel) was performed using Fiji software, with band intensities normalized to the proliferative condition and α-tubulin used as a loading control. Data are presented as mean ± SD (*n* ≥ 3), with individual data points shown. Statistical analysis was conducted using a two-tailed, unpaired t-test. **D**, **E** Left panel: Representative images showing the subcellular localization of DCTPP1 (**D**) or dUTPase (**E**) in proliferating and quiescent cells (cropped from 3D reconstructions). Mitochondria were stained with MitoTracker™ Red (red channel), DCTPP1 or dUTPase (green channel), and nuclei were stained with DAPI (blue channel). Scale bar; 10 µm. Right panel: Analysis of the percentage of relative co-localization of DCTPP1 (**D**) or dUTPase (**E**) with the nucleus (top) and mitochondria (bottom) presented as Manders coefficient. Co-localization was scored from ~30 cells per condition across three independent experiments (*n* = 3), using Fiji software. Unless otherwise indicated, all experiments were carried out on day 10 of quiescence. For panels D and E, statistical analysis was performed using an unpaired two-tailed Student’s t-test.

To investigate pyrimidine requirements in non-replicating cells, we employed a quiescence model based on the human lung fibroblast cell line CCD-34Lu. These cells represent a well-established system for studying cellular quiescence and mitochondrial homeostasis, and they have been extensively used to elucidate mechanisms underlying mitochondrial DNA (mtDNA) maintenance [16]. Cells were grown to confluence, followed by a reduction in fetal bovine serum (FBS) concentration to 0.1%, and cultured under these conditions for 1–2 weeks. As shown in Fig. 1A, after 2 days of serum depletion, less than 1% of cells were in S-phase. Contact inhibition led to a decrease in S-phase population from approximately 10% in proliferative cells to < 2%, with a corresponding accumulation in G1-phase. Further reduction in FBS concentration decreased the S-phase cell population to between 0.15–0.02% (Fig. 1A). Minor changes in cell viability were observed upon induction of quiescence (Fig. 1B), confirming that CCD-34Lu cells halted nuclear DNA replication and cell division while remaining viable.

**Fig. 2:**
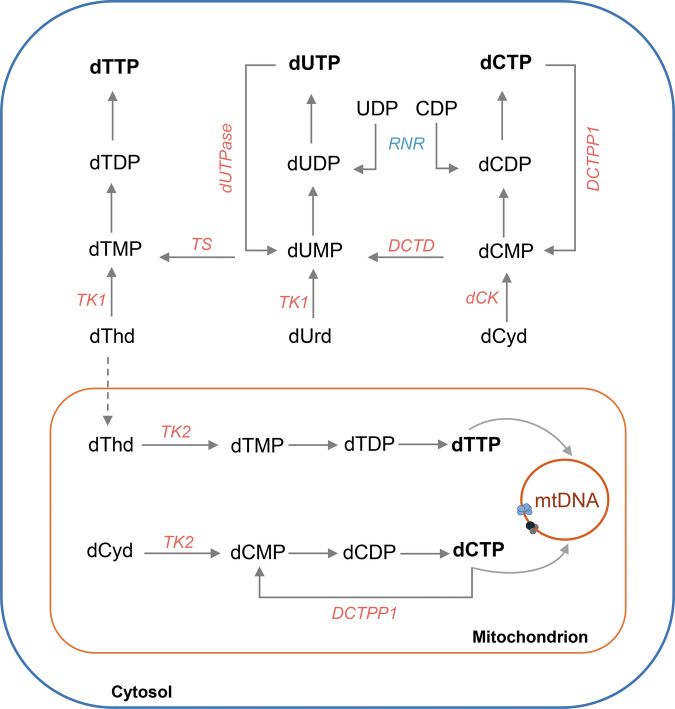
Schematic overview of mitochondrial and cytosolic pyrimidine nucleotide metabolism. Pyrimidine nucleotides required for DNA synthesis (dCTP and dTTP) are produced through de novo and salvage pathways. In the de novo route, CDP and UDP are reduced to their deoxy forms by ribonucleotide reductase (RNR) and subsequently phosphorylated by nucleoside-diphosphate kinases to their triphosphate form. Formation of dTMP is catalysed by thymidylate synthase (TS) via methylation of dUMP, which mainly derives from dCMP deamination through deoxycytidylate deaminase (DCTD). Additional enzymes contribute to nucleotide catabolism: dUTP diphosphatase (dUTPase) hydrolyses dUTP to dUMP, and dCTP pyrophosphatase 1 (DCTPP1) converts dCTP to dCMP. In the salvage pathway, nucleosides released during nucleic acid turnover are phosphorylated by specific kinases: deoxycytidine kinase (dCK) for deoxycytidine and thymidine kinases, TK1 (cytosolic) and TK2 (mitochondrial) for uracil- and thymidine-derived nucleosides.

The quiescent model obtained this way was analysed by western blot for quantification of the expression of several enzymes involved in pyrimidine metabolism. Figure 2 provides a schematic overview of mitochondrial and cytosolic pyrimidine nucleotide metabolism, illustrating the key enzymatic steps and pathways evaluated. As shown in Fig. 1C, we observed a significant reduction in the levels of DCTPP1, thymidylate synthase (TS), and deoxycytidine kinase (dCK), while levels of dCMP deaminase (DCTD) remained unchanged. In line with previous studies [16] thymidine kinase 1 (TK1) was practically undetectable in quiescent cells. Notably, the expression of the nuclear and mitochondrial isoforms of dUTPase was altered in quiescence. The nuclear isoform (n-dUTPase) was reduced by approximately 70–80%, while the mitochondrial isoform (mt-dUTPase) exhibited a two-fold increase. Additionally, the sterile alpha motif and HD-domain containing protein 1 (SAMHD1) was significantly upregulated in quiescent cells, while the levels of p53R2, the small subunit of RNR known to be essential for DNA repair [7] and mitochondrial DNA synthesis [8], remained unaffected. Interestingly, the expression of mitochondrial TK2 was reduced, although its presence in quiescent cells remained detectable.

To further investigate the subcellular localization of pyrimidine-metabolizing enzymes, and to confirm the modulation of these enzymes upon quiescence induction, we conducted immunofluorescence studies (Supplementary Fig. S1). Notably, DCTPP1, which is ubiquitously distributed in proliferating cells [12], was found to predominantly localize to mitochondria in quiescent cells (Fig. 1D). In cycling CCD-34Lu cells, DCTPP1 was primarily cytosolic, accounting for up to 70% of the total enzyme, with the remainder distributed between the nucleus and mitochondria. In contrast, in quiescent cells, DCTPP1 was predominantly localized to the mitochondria, representing approximately 80% of total DCTPP1, with much smaller amounts present in the cytosol (10–20%) and nucleus (5–10%). Co-localization analysis using the mitochondria-specific probe MitoTracker™ Red CMXRos confirmed that DCTPP1 is predominantly associated with mitochondria in non-replicating cells.

Regarding dUTPase, another important NTP pyrophosphatase, immunolocalization analysis revealed significant changes in the distribution of the two isoforms, n-dUTPase and mt-dUTPase, upon quiescence induction. In cycling cells, n-dUTPase was predominantly localized to the nucleus, while in quiescent cells, the remaining staining corresponded primarily to the mitochondrial isoform, in agreement with the western blot data (Fig. 1E).

### Impact of DCTPP1 depletion on pyrimidine metabolism enzyme expression and cell viability in proliferating cells

**Fig. 3:**
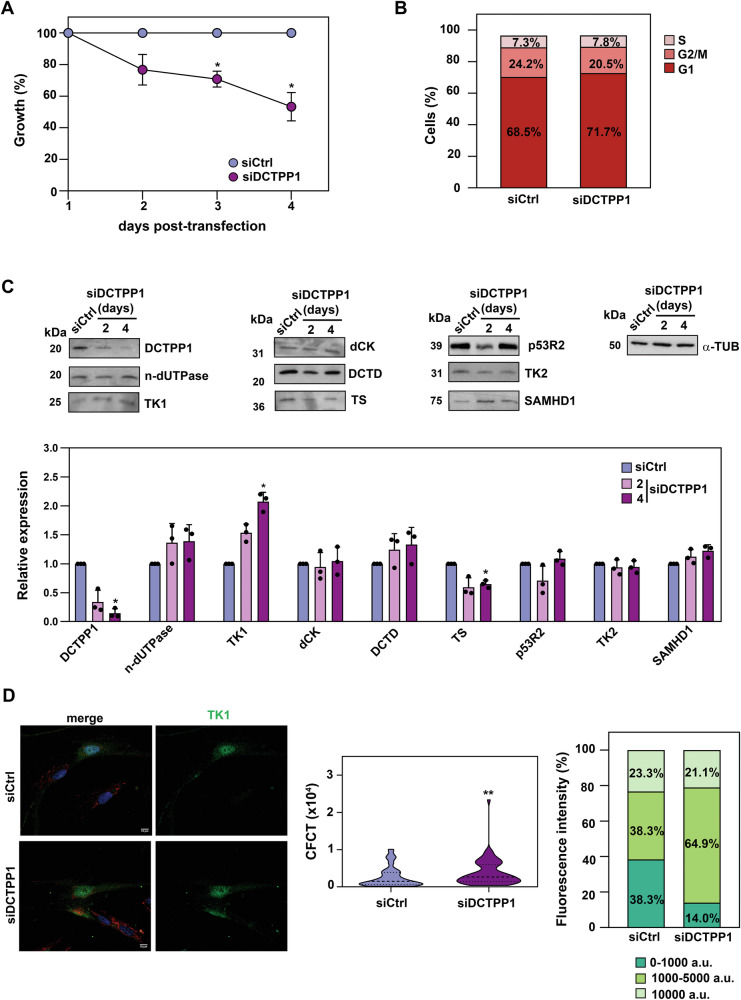
Impact of DCTPP1 depletion on enzyme expression and cell viability in proliferating cells. **A** Cumulative proliferation growth curve of CCD-34Lu cells transfected with DCTPP1 (siDCTPP1) or control (siCtrl) siRNAs. Each data point represents the mean from three biological replicates, with values shown as mean ± S.D. (*n* ≥ 3). A two-way ANOVA was performed (F_(3,12)_ = 33.16; *p* < 0.001), followed by Dunnett’s multiple comparison correction. **B** Cell cycle profile of DCTPP1-siRNA transfected cells after 4 days of transfection, expressed as percentage of cells in each cell cycle phase. Values are mean ± S.D. (*n* ≥ 3). A two-way ANOVA was performed with Sidak’s post hoc test to compare siDCTPP1 transfected cells (day 4) versus control cells (siCtrl), n.s. *p* < 0.12. **C** Western blot analysis of key pyrimidine metabolism enzymes in DCTPP1-depleted cells at the indicated time points (upper panel). Quantification of Western blot bands from siCtrl or siDCTPP1 cycling cells (lower panel). siRNA-transfected cells were used as controls, and anti-α-tubulin was employed to normalize protein levels. Band intensities were quantified using ImageJ/Fiji software. Only the relevant sections of the full-length blots are shown, with original blots provided in the Supplementary data. Values represent means ± S.D. (*n* ≥ 3), individual data points shown. Statistical analysis was performed using the Kruskal–Wallis test, followed by Dunn’s multiple comparisons correction. **D** Immunofluorescence analysis of TK1 upon DCTPP1 down-regulation. Left panel, representative images of TK1 expression (green). Nuclei were stained with DAPI (blue) and mitochondria with MitoTracker™ Red (red). Scale bar, 10 µm; middle panel, quantification of total TK1 fluorescence intensity expressed as corrected total cell fluorescence (CTCF, x10^4^) and distribution of fluorescence intensity levels as percentages (right panel), stratified into low (0-1000 a.u.), medium (1000-5000 a.u.) and high intensity (5000–10,000 a.u.). (Arbitrary Units, a.u.). Approximately 60 cells per condition were analysed across two independent experiments. Statistical analysis of TK1 CTCF was performed using an unpaired, two-tailed Mann–Whitney U test.

Given the cell-type-dependent role of DCTPP1 in DNA replication and cell survival [12–14], we sought to investigate the effects of DCTPP1 down-regulation in proliferating CCD-34Lu cells. DCTPP1 depletion was induced via transient transfection with small interfering RNA (siRNA), achieving a 70–80% reduction in protein levels, with DCTPP1 expression remaining low throughout the study period. As shown in Fig. 3A, DCTPP1 depletion caused defects in cell proliferation, with a 40–50% reduction in cell growth observed by day 4 post-transfection, relative to control cells. Cell cycle analysis (Fig. 3B) revealed no significant alterations in cell cycle distribution, suggesting that the observed proliferation defects were not associated with major changes in cell cycle progression.

We next assessed the impact of DCTPP1 depletion on the expression of key enzymes involved in pyrimidine metabolism, using western blot analysis (Fig. 3C). As shown in Fig. 3C, levels of n-dUTPase, dCK, TK2, p53R2, DCTD and SAMHD1 were unaffected by DCTPP1 silencing. However, TK1, a key enzyme in thymidine salvage, was upregulated approximately two-fold in DCTPP1-deficient cells. In contrast, TS, responsible for de novo thymidylate synthesis, exhibited a moderate reduction in expression. Immunofluorescence analysis confirmed the increase in TK1 levels (Fig. 3D), providing further support for the upregulation of salvage pathways in response to DCTPP1 depletion. Collectively, these findings suggest that DCTPP1 depletion enhances TK1-mediated salvage mechanisms without significantly altering other pyrimidine-metabolizing enzymes, as evidenced by both western blot and immunofluorescence studies (Fig. 3C, D and supplementary Fig. S2).

### Impact of DCTPP1 down-regulation on the expression of pyrimidine metabolism enzymes in quiescent cells

In non-replicative cells, both de novo dNTP biosynthesis and cytosolic salvage pathways are down-regulated due to the cessation of nuclear DNA replication. However, active mitochondrial DNA (mtDNA) replication and repair remain. Under these conditions, the maintenance of the mitochondrial dNTP pool relies heavily on the mitochondrial salvage pathway [7]. We hypothesized that DCTPP1, as a potential regulator of dCTP pool homeostasis, might play a particularly crucial role in non-dividing cells, contributing to the fidelity of mtDNA replication.

**Fig. 4:**
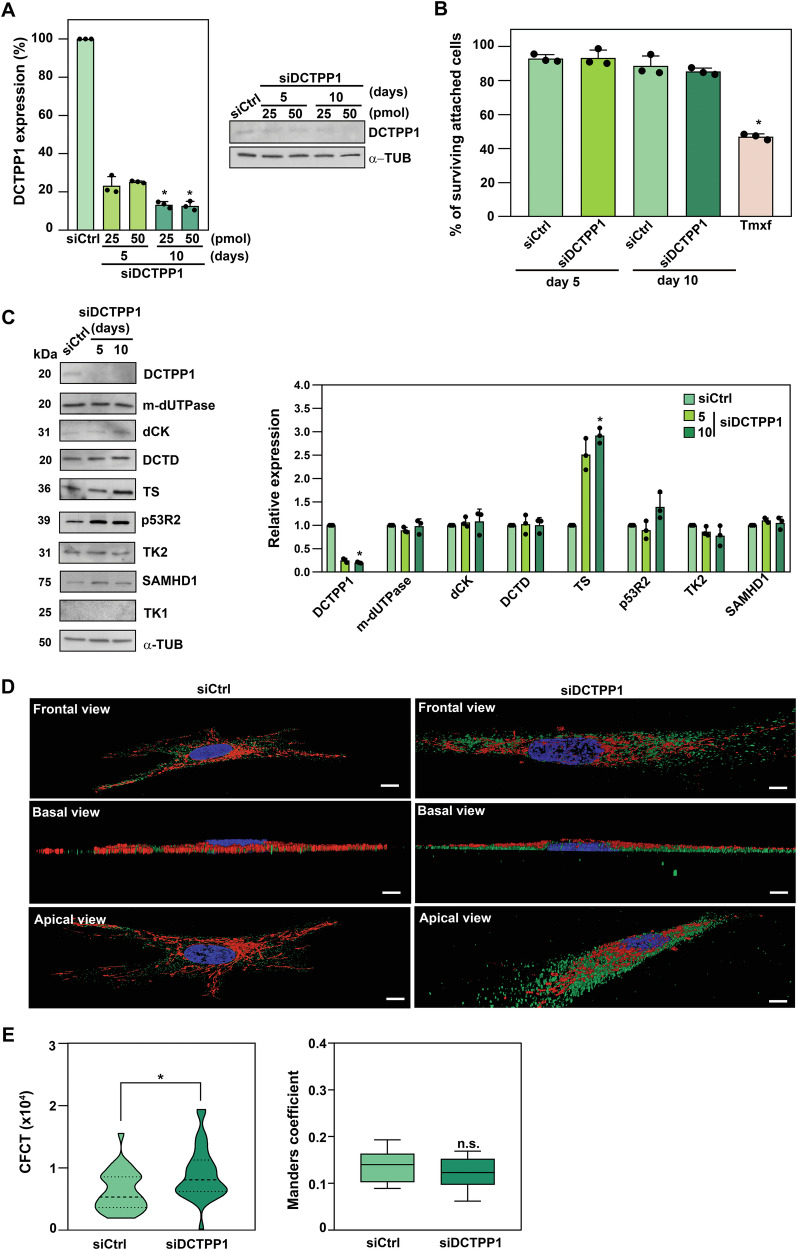
DCTPP1 knockdown in CCD-34Lu quiescent cells. **A** Western blot analysis upon DCTPP1 depletion in CCD-34Lu quiescent by transfection with siDCTPP1 or siCtrl for 5 and 10 days, using 50 or 25 pmol of siRNA (right panel). Control siRNA-transfected cells were used as a control and anti-α-tubulin was used as a loading control. Western blot quantification (left panel). Values are mean ± S.D. (*n* ≥ 3). **B** Crystal Violet assay assessing cell viability in DCTPP1 or control siRNA-transfected quiescent cells, with tamoxifen-treated cells (Tmxf, 50 µM 12 h) as positive control for cell death. Values are mean ± S.D. (*n* ≥ 3). **C** Western blot comparison of pyrimidine metabolism enzyme expression levels in quiescent cells transfected with siCtrl or siDCTPP1, normalized to siCtrl-transfected cells and anti-α-tubulin were used as a loading control, at the indicated time points. Only relevant sections of full-length blots are shown, with original blots provided in Supplementary File. Results are shown as means ± SD (*n* ≥ 3), with individual data points included. Statistical analysis was performed using the One-way ANOVA Kruskal Wallis test with Dunn’s multiple comparison correction for (**A–C**). **D** Representative high-resolution confocal microscopy images of the subcellular localisation of thymidylate synthase (TS, green) upon siCtrl (left panel) or siDCTPP1 transfection (right panel). Nuclei were stained with DAPI (blue), and mitochondria were stained with MitoTracker™ Red (red). 3D reconstructions are shown from frontal, basal, or apical views. Scale bar; 10 µm. **E** Quantification of total TS fluorescence intensity (corrected total cell fluorescence, CTCF) and TS mitochondrial colocalization (Manders coefficient) in approximately 30 cells scored per condition (siCtrl or siDCTPP1) using FIJI software (from three independent replicates; *n* = 3). Statistical analysis was performed using an unpaired two-tailed Student’s *t*-test.

To investigate the role of DCTPP1 in quiescent cells, CCD-34Lu cells were cultured to contact inhibition and then induced into quiescence by serum starvation. Simultaneously, cells were transfected with either siRNA targeting DCTPP1 or a control siRNA. DCTPP1 depletion was confirmed by western blot, showing an ~ 80% reduction in protein levels (Fig. 4A). Cell viability assays using crystal violet staining indicated that DCTPP1 depletion did not significantly impact the percentage of viable attached cells in quiescent cultures, at either 5 or 10 days post-transfection (Fig. 4B).

We next assessed the impact of DCTPP1 depletion on enzymes involved in dNTP biosynthesis and salvage pathways. As shown in Fig. 4C, the expression of TS, an enzyme responsible for converting deoxyuridine monophosphate (dUMP) to deoxythymidine monophosphate (dTMP), increased approximately three-fold in DCTPP1-deficient quiescent cells. Additionally, a modest but significant increase in p53R2 levels was observed. Immunofluorescence analysis confirmed these findings (Supplementary Fig. 3), with high-resolution confocal microscopy (Fig. 4D) showing elevated TS levels in DCTPP1-depleted cells, predominantly localized in the cytosol. Consistent with this, TS did not exhibit substantial co-localization with mitochondria, as reflected by the low Manders colocalization coefficients, which did not differ between control and DCTPP1-depleted cells (Fig. 4E).

### Down-regulation of DCTPP1 perturbs the dNTP pool in human lung fibroblasts

**Fig. 5:**
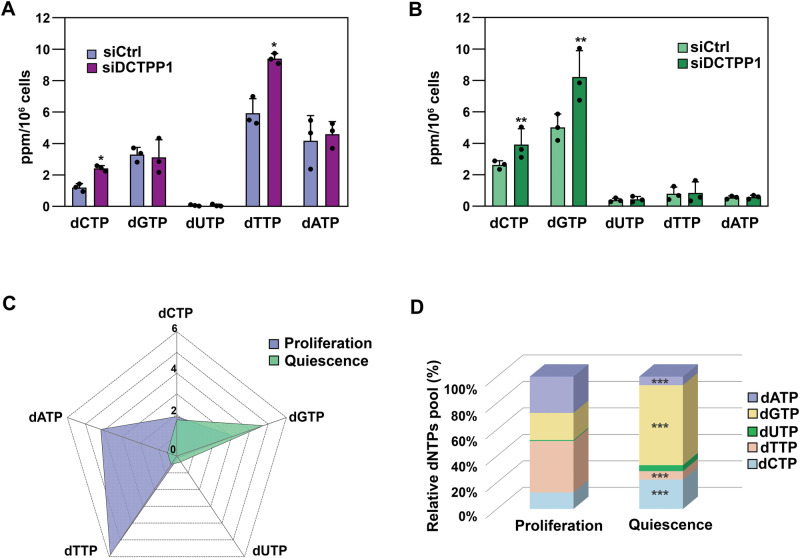
dNTP pool disturbances in CCD-34Lu cells. **A** Pyrimidine dNTP levels measured by the polymerase-based assay in DCTPP1-deficient proliferative cells upon 4 days post down-regulation and **B** in quiescent CCD-34Lu cells upon 10 days of DCTPP1 depletion. Data are presented as mean concentrations with error bars indicating ± SD from three independent experiments with triplicate technical replicates and individual values are shown. Statistical analyses in (**A**) and (**B**) were performed using a two-tailed Student’s *t*-test (confidence level 95%). **C** and **D** present a comparison of dNTP pools between proliferative and quiescent CCD-34Lu cells. In panel D, a two-way ANOVA followed by Sidak’s multiple comparison test was applied.

To evaluate the impact of DCTPP1 depletion on nucleotide homeostasis, we analysed the pyrimidine dNTP pools in both proliferative and quiescent CCD-34Lu cells (Fig. 5). In proliferating cells, consistent with the proposed role of DCTPP1 [12, 13], depletion led to a significant increase in the dCTP pool, rising from 1.19 pmol/10^6^ cells in control cells to 2.5 pmol/10^6^ cells. Additionally, DCTPP1-depleted proliferative cells exhibited a notable increase in dTTP levels, rising from 4.9 pmol/10^6^ in control cells to 9.4 pmol/10^6^ (Fig. 5A).

Interestingly, both dCTP and particularly dGTP were found to be the most abundant dNTPs in quiescent cells and the depletion of DCTPP1 led to a further expansion of these dNTPs. Specifically, dCTP levels increased from 1.82 pmol/10^6^ in control cells to 3.5 pmol/10^6^ (Fig. 5B) while dGTP levels doubled, from 4.75 pmol/10^6^ (constituting ~ 60% of the total dNTP pool) in control cells to 9.5 pmol/10^6^ (80% of the total dNTP pool) in DCTPP1-depleted cells (Fig. 5B). This striking increase in dGTP and dCTP underscores the contribution of DCTPP1 to maintaining the nucleotide balance in non-replicative cells and supports its potential involvement in mitochondrial DNA replication and repair, where accurate nucleotide homeostasis is essential.

We further compared dNTP pools between proliferative and quiescent CCD-34Lu cells (Fig. 5C). As previously reported [3], dNTP levels were generally higher in proliferating cells, in line with the demand for elevated dNTPs during DNA replication. Specifically, dTTP and dATP together accounted for approximately 65% of the total dNTP pool in proliferative cells, whereas dGTP was the predominant dNTP in quiescent cells (Fig. 5D).

### Consequences of DCTPP1 depletion on mitochondrial function and mtDNA stability

**Fig. 6:**
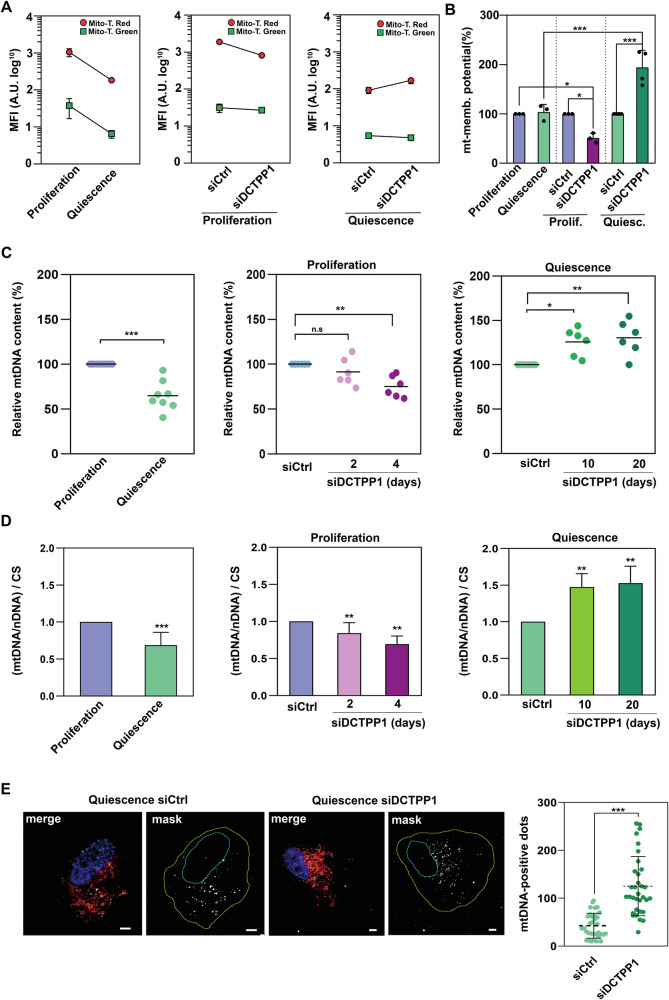
Impact of DCTPP1 depletion on mitochondrial function and genome integrity. **A** Mitochondrial function was evaluated using dual staining with MitoTracker™ Green (mitochondrial mass) and MitoTracker™ Red (membrane potential), followed by flow cytometry analysis. Data are expressed as MFI-corrected median fluorescence intensity (MFI), in arbitrary units (a.u., log10 scale), across all experimental conditions. Error bars represent standard deviation (S.D.) from three biological replicates, with cells cultured in separate wells/dishes within a single experiment. **B** Mitochondrial membrane potential was normalized and expressed as a percentage by calculating the ratio of fluorescence intensity of MitoTracker™ Red (membrane potential-sensitive dye) to that of MitoTracker™ Green. Data are presented as means ± S.D. (*n* ≥ 3), with individual data points included. A two-way ANOVA was performed, followed by Tukey’s post hoc test, revealing a significant interaction effect (F_(2,14)_ = 36.40, *p* < 0.001). **C** mtDNA copy number was determined by real-time quantitative PCR, measuring the relative mitochondrial DNA content (mtDNA/nDNA) under various conditions, including proliferation versus quiescence and siRNA depletion (siCtrl vs. siDCTPP1) in both proliferation and quiescence states, at the indicated time points. Individual data points are shown (*n* ≥ 6). **D** mtDNA copy number normalized to citrate synthase (CS) activity (nmol/min × mg protein, see Supplementary Fig. 5) is shown, with each column representing the mean of (mtDNA/nDNA)/CS values ± S.D. (*n* ≥ 3). Statistical significance was assessed using an unpaired Student’s *t*-test for (**C**) and (**D**) (left; proliferation vs. quiescence), and a Kruskal–Wallis test followed by Dunn’s multiple comparisons correction for the right panels in (**C**) and (**D**) (siCtrl vs. siDCTPP1 in proliferation and quiescence, respectively). **E** Representative in situ images of mtDNA in quiescent cells transfected with either control (siCtrl) or DCTPP1-targeted (siDCTPP1) siRNA for 10 days. Nuclei were stained with DAPI (blue), mitochondrial signal was marked with MitoTracker™ Red (red), and in situ mtDNA-positive dots are shown in grey (far-red). A mask was applied to quantify mtDNA-positive dots, as shown in the confocal images. Left panel: Quantification of mtDNA-positive dots using Fiji software, with individual data points presented (*n* ≥ 30). Statistical analysis was performed using an unpaired, two-tailed Mann–Whitney U test. Scale bar: 10 µm.

To assess the impact of DCTPP1 depletion on mitochondrial function, we employed fluorescence-based dyes to evaluate mitochondrial activity. Specifically, we utilized MitoTracker™ Red CMXRos and MitoTracker™ Green. MitoTracker™ Red is sensitive to the mitochondrial membrane potential and fluorescence decreases upon depolarization. In contrast, MitoTracker™ Green labels the mitochondrial mass independently of membrane potential, serving as a direct measure of mitochondrial abundance [17]. Flow cytometry quantification was performed for each marker and expressed as mean fluorescence intensity (MFI, arbitrary units) (Fig. 6A) [18]. In addition, the mitochondrial membrane potential was normalized considering mass and expressed as the ratio of fluorescence intensity of MitoTracker™ Red (membrane potential-sensitive dye) versus that of MitoTracker™ Green (Fig. 6B). Induction of quiescence induces a decrease in mitochondrial mass and membrane potential yet normalization indicates the preservation of mitochondrial function (Fig. 6A, B). On the other hand, while in replicating cells DCTPP1 depletion affects moderately mitochondrial function, in quiescent cells a decrease in DCTPP1 exerts a clearly protective role (Fig. 6B).

We therefore hypothesized that by contributing to the maintenance of the dCTP/dTTP ratio in non-dividing cells, DCTPP1 may play a critical role in mtDNA replication and repair thus influencing mitochondrial integrity and function. To investigate this, we measured the effect of DCTPP1 depletion on mtDNA levels using two methodological approaches: real-time quantitative PCR (qPCR) (Fig. 6C, D) [19, 20] and a monoclonal anti-DNA antibody labelling technique, which allows for mtDNA visualization in situ (Fig. 6E and Supplementary Fig. 4) [21, 22]. To accurately assess mtDNA content relative to mitochondrial abundance, qPCR data were normalized not only to a nuclear-encoded reference gene but also to citrate synthase (CS) activity, a well-established biochemical marker of mitochondrial mass (Fig. 6D and Supplementary Fig. 5). This dual normalization strategy helps to distinguish whether changes in mtDNA levels reflect genuine alterations in mitochondrial DNA maintenance or are secondary to shifts in overall mitochondrial content [23]. Interestingly, both qPCR (Fig. 6C, D) and antibody-based determinations (Fig. 6E) evidenced that DCTPP1-depleted cells in the post-replicative state exhibit an increase in mtDNA copy number compared to control cells transfected with siCtrl.

### DCTPP1 suppression in quiescent cells reverts mitochondrial DNA depletion induced by thymidine overloading

Mitochondrial neurogastrointestinal encephalomyopathy (MNGIE) is a rare disorder caused by thymidine phosphorylase (TP) deficiency [24], which results in an imbalance of mitochondrial dNTPs and an accumulation of mtDNA mutations [25–27]. Previous studies regarding the causes for mtDNA depletion in MNGIE suggested that an increase in thymidine triphosphate (dTTP) limits the availability of deoxycytidine triphosphate (dCTP), essential for mtDNA replication. Indeed, supplementation with deoxycytidine has been shown to alleviate the effects of this imbalance [28]. Overall, our results suggest that DCTPP1 plays a role in mtDNA integrity and that its depletion may protect mtDNA. Modulating DCTPP1 activity, therefore, may present a novel therapeutic strategy for MNGIE and similar mitochondrial DNA depletion syndromes where dCTP is crucial for efficient mtDNA replication.

**Fig. 7:**
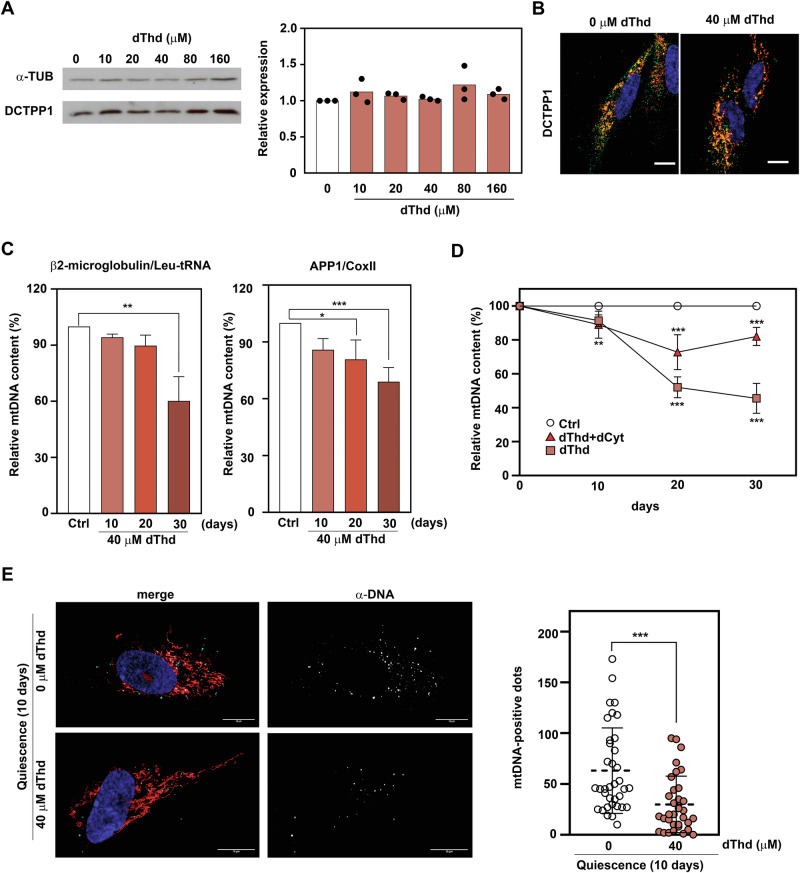
An in vitro MNGIE model: quiescent cell exposed to prolonged thymidine overload. **A** DCTPP1 western blot analysis was performed in CCD-34Lu quiescent cells after thymidine overload at the indicated dThd concentrations (0-160 μM). Western blot quantification was performed using Fiji software and α-tubulin as loading control (right panel). Original blots are presented in Supplementary data. **B** Representative immunofluorescence images of DCTPP1 staining in the presence or absence of dThd overload are shown (left panel). Showing nuclei stained with DAPI (blue channel), mitochondrial signal labeled with MitoTracker™ Red (red channel), and DCTPP1 staining (green channel). **C** mtDNA copy number was quantified by qPCR after dThd overload (40 µM) at the indicated time points. The mtDNA copy number (mtDNA/nDNA) was normalized to control conditions without dThd. Data are presented as mean ± SD (*n* ≥ 3). Statistical analysis was performed using a Kruskal–Wallis test, followed by Dunn’s multiple comparisons correction for (**A**) and (**C**). **D** Reversal of dThd overload-induced mtDNA defects was assessed after dCyt (40 µM) administration, measured by qPCR at indicated times. Each point represents the mean from three biological replicates (*n* = 3). Statistical analysis was conducted using a two-way ANOVA (F_(6, 60)_ = 45.02, *p* < 0.001), with Dunnett’s post hoc test applied for multiple comparisons. **E** Representative immunofluorescence images of mtDNA-positive dots (left panel) are shown; red channel: mitochondria marker (MitoTracker™ Red), blue channel: nucleus staining (DAPI), and grey (far-red channel): mtDNA staining with anti-DNA antibody, clone AC-30-10 (CBL186), in the presence or absence of dThd (40 µM). Right panel: Quantification of mtDNA-positive dots was performed using Fiji software on 30 individual cells per condition, across three independent experiments (*n* = 3). Statistical analysis was conducted using an unpaired, two-tailed Student’s t-test. Scale bar; 10 µm.

To explore this hypothesis, we used an established in vitro MNGIE model, which involves thymidine (dThd) overloading to mimic thymidine phosphorylase defects [16]. We first assessed DCTPP1 expression and distribution in this cell model. Hence, western blotting and immunofluorescence analyses were performed on CCD-34Lu cells supplemented with high concentrations of thymidine during 10 days of quiescence. No significant changes in DCTPP1 expression were observed as determined by western blot (Fig. 7A). In addition, thymidine overloading did not affect the intracellular distribution of DCTPP1 (Fig. 7B)

We then evaluated the impact of thymidine overload (40 μM dThd) on mtDNA integrity using qPCR in quiescent CCD-34Lu cells. The results revealed that thymidine overload led to a significant reduction in mtDNA copy number after 20 days of incubation (Fig. 7C), consistent with previous studies [29]. Notably, supplementation with deoxycytidine (40 µM) reversed the deleterious effects of thymidine overload on mtDNA, as shown in Fig. 7D. We further visualized mtDNA in situ using the monoclonal anti-DNA antibody [21, 22]. High-resolution confocal microscopy and subsequent quantification of mtDNA-positive dots confirmed the decrease in mtDNA copy number following thymidine overload (Fig. 7E), validating our in vitro model for MNGIE-induced mtDNA depletion.

**Fig. 8:**
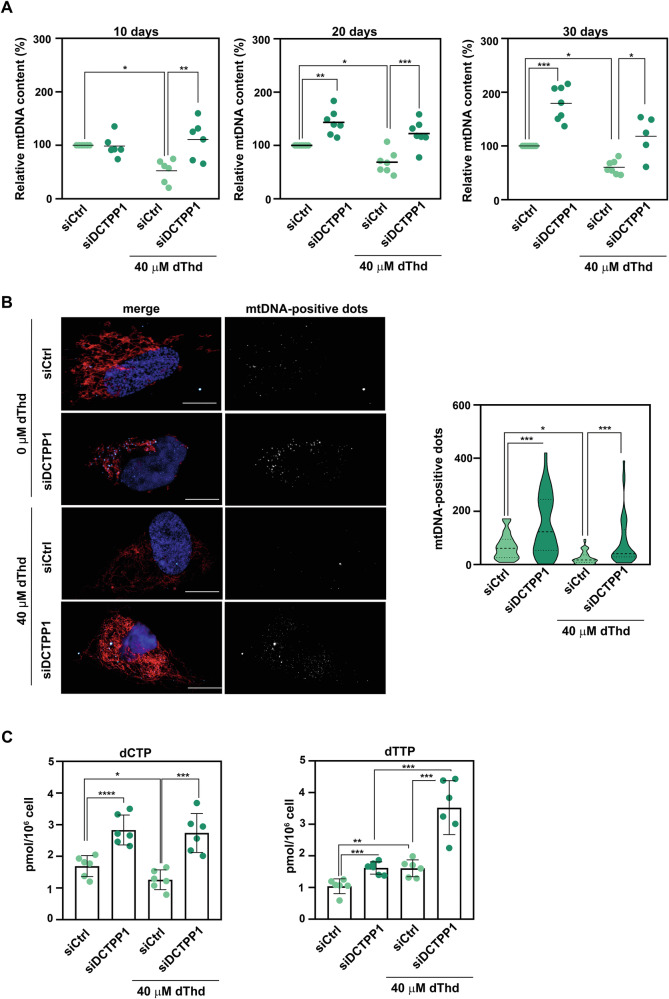
Potential role of DCTPP1 in the treatment of mitochondrial DNA depletion syndromes such as MNGIE. **A** mtDNA copy number was determined by real-time quantitative PCR, measuring the relative mitochondrial DNA content (mtDNA/nDNA) upon DCTPP1 depletion by siRNA in presence or absence of thymidine overload (40 µM dThd) at indicated time points. Control siRNA-transfected cells in absence of dThd overload were used as a control. Individual data points are shown (*n* ≥ 6), with statistical significance determined using a two-way ANOVA followed by Tukey’s multiple comparisons correction. **B** Representative immunofluorescence images of mtDNA-positive dots (left panel) following DCTPP1 depletion, in the presence or absence of dThd overload. Control siRNA-transfected cells are included as internal controls. Mitochondria are labeled with MitoTracker™ Red (red channel), nuclei are stained with DAPI (blue channel), and mtDNA is visualized in grey using an anti-DNA antibody (clone AC-30-10, CBL186; far-red channel). dThd was used at 40 µM. Scale bar; 10 µm. Right panel: Quantification of mtDNA-positive dots was performed using Fiji software ( ≥ 30 cells per condition from three independent experiments, *n* = 3). **C** dNTP pools alteration, particularly dCTP (left panel) and dTTP (right panel), in response to thymidine overload was analized. Cells were transient silencing for Control or DCTPP1-target siRNA during 20 days and dNTP levels were measured by a polymerase-based assay. Data are presented as mean concentrations, with error bars representing mean ± SD from three independent experiments with triplicate technical replicates. Individual values are also shown. Statistical analysis was performed using an unpaired two-tailed Student’s t -test for (**B**,**C**).

Next, we examined the role of DCTPP1 in this MNGIE cell model by assessing mtDNA levels and the dCTP/dTTP nucleotide pools in the presence or absence of thymidine overload. Interestingly, both PCR analysis (Fig. 8A) and in situ mtDNA staining (Fig. 8B) revealed that DCTPP1 depletion increased mtDNA copy numbers compared to controls in quiescent cells. This protective effect was also observed in the MNGIE model, where DCTPP1 depletion helped preserve mtDNA levels despite thymidine overload. The in situ mtDNA staining also corroborated this finding, showing increased mtDNA levels in DCTPP1-depleted cells compared to controls after 20 days of thymidine overload (Fig. 8B).

To investigate the underlying mechanism of mtDNA content modulation, we analysed the dNTP pools, particularly dCTP and dTTP, in response to thymidine overload. In line with previous reports [29, 30] we found that, in control cells, 20 days of thymidine supplementation (40 µM), induces an expansion of the dTTP pool, while the dCTP pool was slightly but significantly decreased (Fig. 8C). Importantly, transient silencing of DCTPP1 resulted in a major expansion of the dCTP pool in both non-treated and thymidine overloaded cells. These findings suggest that DCTPP1 depletion, even under thymidine overload conditions, can expand the dCTP pool, potentially explaining its protective role in maintaining mtDNA integrity by counteracting the harmful effects on mtDNA replication.

In summary, DCTPP1 depletion appears to mitigate the damaging effects of thymidine overload on mtDNA levels, likely through modulation of the dCTP/dTTP balance. These findings support the potential of DCTPP1 modulation as a therapeutic strategy for MNGIE and other mitochondrial DNA depletion syndromes, where the maintenance of adequate dNTP pools is crucial for mitochondrial function and genome stability.

### The DCTPP1 inhibitor TH1217 reverts mtDNA depletion

To assess the therapeutic potential of DCTPP1 as a druggable target in mitochondrial DNA depletion syndromes (MDSs), we examined whether small-molecule inhibition of DCTPP1 could modulate mitochondrial nucleotide homeostasis and promote mtDNA maintenance. For these studies, we employed TH1217 (ZINC1775962367; HY-135909 MCE), a potent and selective DCTPP1 inhibitor with an IC₅₀ of 47 nM [31]. TH1217 exhibited moderate cytotoxicity, with an EC₅₀ of ~28.5 μM at 72 h in proliferating CCD-34Lu cells, supporting its suitability for probing DCTPP1 function in intact cellular systems.

**Fig. 9:**
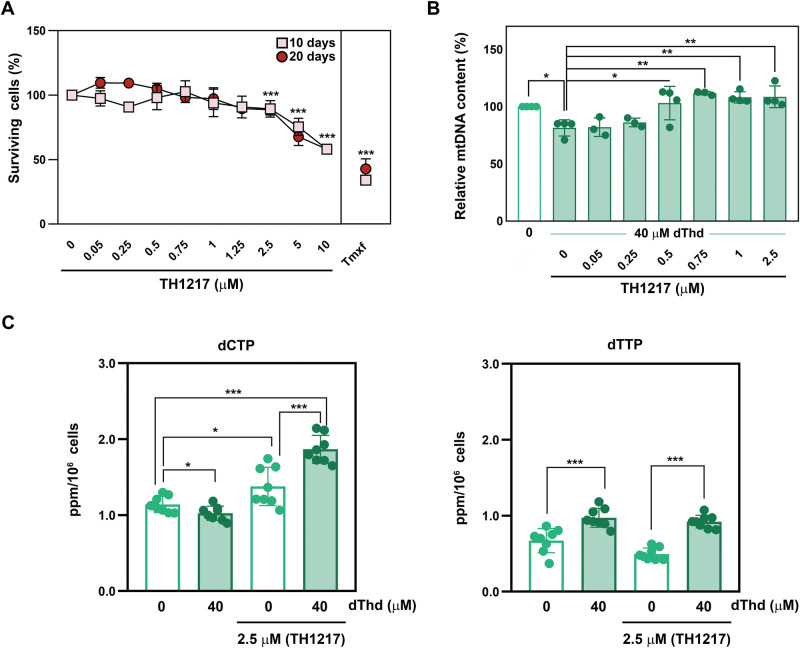
Treatment with a DCTPP1 inhibitor restores mtDNA content and modulates mitochondrial dNTP pools. **A** Cytotoxicity profile of the DCTPP1 inhibitor TH1217 in quiescent CCD-34Lu fibroblasts over 10–20 days at concentrations ranging from 0.5–10 µM. Tamoxifen (50 µM, Tmxf) was used as a positive toxicity control. Statistical analysis was conducted using a two-way ANOVA (F_(11, 59)_ = 2.55, *p* = 0.010), with Dunnett’s post hoc test applied for multiple comparisons. **B** mtDNA copy number in CCD-34Lu cells subjected to dThd overload (40 μM, 20 days) with or without TH1217 (0.05–2.5 µM), showing rescue of mtDNA depletion. Ordinary one-way ANOVA with Dunnett’s post hoc test was applied for statistical analysis of the effect of TH1217 treatment in thymidine treated cells while the effect of thymidine overloading alone on mtDNA versus non-treated controls was analysed using the Student’s t-test **C** dCTP (left panel) and dTTP (right panel) pools in response to 20 days of TH1217 treatment (2.5 μM) in the presence or absence of thymidine overload (40 μM, 20 days) was analysed. Data are presented as mean concentrations, with error bars representing mean ± SD from three independent experiments. Individual values are also shown. Statistical analysis was performed using an unpaired two-tailed Student’s t -test.

We next evaluated the cytotoxicity profile of TH1217 in quiescent CCD-34Lu fibroblasts. A significant loss of viability was detected only at concentrations above 5 µM, after prolonged exposure for 10 or 20 days (Fig. 9A). The impact of TH1217 on mitochondrial nucleotide homeostasis and genome maintenance was therefore assessed at concentrations lower than 5 µM in the in vitro MNGIE model, using dThd overloading (40 μM). Interestingly, TH1217 administered at concentrations ranging from 0.5 to 2.5 μM led to a measurable increase in the mtDNA relative copy number, significantly mitigating the depletion induced by excess dThd (Fig. 9B).

In addition, and consistent with the effect on mtDNA content, treatment with 2.5 µM TH1217 induces an expansion of the dCTP pool notably in thymidine overloaded cells (Fig. 9C). These observations further support the connection between DCTPP1 modulation, dCTP pools and mtDNA depletion.

## Discussion

Disruptions in mitochondrial nucleotide metabolism can lead to MDSs, impairing mitochondrial function and contributing to a range of pathologies [24, 32, 33]. In proliferating cells, imbalances in mitochondrial dNTP pools can be compensated by the import of nucleotides from the cytosol [8, 29]. However, in quiescent cells, where mitochondrial DNA replication remains active despite the absence of cell division, the preservation of dNTP homeostasis depends mainly on the salvage pathway. Hence, perturbations in dNTP pools can affect mtDNA replication and mitochondrial function [34].

CCD-34Lu cells, a well-established model for studying cellular quiescence [16], were used to explore the role of DCTPP1, a dNTP pyrophosphatase with high affinity for dCTP and a prominent role in the balance of canonical pyrimidine nucleotide pools [12, 13]. We examined how DCTPP1 influences dNTP homeostasis and mtDNA maintenance under quiescent conditions. We provide a detailed study on the behaviour of several pyrimidine metabolism enzymes upon induction of quiescence and the modifications that arise upon DCTPP1 modulation in this cellular context. Upon induction of quiescence, several enzymes involved in cytosolic de novo synthesis and salvage, including DCTPP1, are down-regulated. However, the remaining DCTPP1 is primarily located in mitochondria, suggesting a potential role in modulating dCTP levels in this organelle in the absence of cell division.

Depletion of DCTPP1 in cycling CCD-34Lu cells results in an expansion of the dCTP and dTTP pools, supporting the notion that DCTPP1 not only maintains dCTP homeostasis but also contributes to dTTP synthesis by providing dCMP, as previously described for other cell types [13]. We now extend these findings to quiescent cells, where transient silencing of DCTPP1 also results in elevated dCTP levels. This observation is particularly relevant given the importance of precise dNTP ratios for mtDNA replication fidelity and mitochondrial function.

Interestingly, DCTPP1 depletion in quiescent cells also led to an unexpected increase in the dGTP pool. It has been reported that dGTP is largely overrepresented among other dNTPs in mitochondria [30] and that a vast majority of mitochondrial dGTP is tightly bound to NDUFA10, an accessory subunit of complex I of the mitochondrial respiratory chain [35]. While the mechanism underlying dGTP accumulation following DCTPP1 depletion in quiescent cells remains unclear, this observation further underscores the importance of regulating mitochondrial dGTP levels. The accumulation of dGTP relative to other dNTPs in non-proliferative tissues, including neurons, has led to the hypothesis that dGTP may serve non-canonical metabolic functions, potentially acting as a signalling molecule or allosteric regulator, beyond its established role in DNA synthesis [36].

Having established the crucial participation of DCTPP1 in modulating dNTP content in quiescent cells, we next examined its impact on mtDNA integrity. By using different methodologies, we establish that down-regulation of DCTPP1 in quiescent cells increases the relative mtDNA content. We propose that the expansion of the dCTP and dGTP pools facilitates replication helping to counteract mtDNA depletion driven by dNTP imbalances.

Imbalances in the mitochondrial pool of dNTPs are well-documented contributors to mtDNA instability and disease. Indeed, there are genetic defects that affect mitochondrial replication and are at the origin of various pathologies. Mutations in TK2, DGUOK, and p53R2 severely influence the supply of dNTPs in differentiated non-replicating cells, leading to various forms of MDSs [34]. Specifically, MNGIE, an ultra-rare disease caused by mutations in the *TYMP* gene encoding thymidine phosphorylase (TP) [24], leads to severe dNTP pool imbalance and the accumulation of point mutations and deletions in mtDNA [26, 27], a phenotype that exemplifies the relationship between mutagenesis and alterations in DNA precursor pools. Importantly, González-Vioque et al. [28]. proposed an explanation for the mtDNA depletion observed in MNGIE, suggesting that mitochondrial replication is not affected by the accumulation of nucleosides per se, but rather by the secondary depletion of dCTP stemming from an increase in dTTP pools, thus limiting its availability for mtDNA biosynthesis. Their work further showed that deoxycytidine supplementation can rescue mtDNA levels, highlighting the central role of dCTP availability. Using thymidine overloading as an in vitro model of MNGIE we now demonstrate, in a similar fashion to deoxycytidine supplementation, that DCTPP1 downregulation exerts a protective effect. Specifically, DCTPP1 silencing leads to dCTP pool expansion and rescues mtDNA depletion.

DCTPP1 has been identified as a highly suitable and druggable target for small-molecule inhibition, and several studies have explored its potential in cancer therapy [31, 37, 38]. A variety of chemical scaffolds have been reported to exhibit inhibitory activity, providing valuable guidance for the design of next-generation compounds. Notably, we show here that pharmacological inhibition of DCTPP1 also modulates mitochondrial dNTP pools and restores mtDNA copy number, thereby opening new avenues for therapeutic intervention of mtDNA depletion syndromes. These findings suggest that therapeutic modulation of DCTPP1 may help restore dNTP balance in MDSs characterized by dCTP deficiency, such as MNGIE. By fine-tuning the dCTP/dTTP ratio, DCTPP1 inhibition could support mtDNA replication and stability under conditions of nucleotide imbalance.

In summary, we identify a previously unrecognized role for DCTPP1 in regulating dNTP pools and maintaining mtDNA integrity in quiescent cells. Our findings point to DCTPP1 as a key modulator of nucleotide metabolism and a potential therapeutic target for mitochondrial DNA depletion syndromes in which dCTP availability is compromised.

## Materials And Methods

### Cell culture

Human lung fibroblasts (CCD-34Lu cells) were obtained from ATCC® (CRL-1491, Manassas, VA, USA). The cell line was authenticated by short tandem repeat (STR) profiling by ATCC prior to purchase and was routinely tested and confirmed to be negative for mycoplasma contamination before and during experimental use. Cells were cultured in T-75 flasks at 37 °C with 5% CO_2_ in minimum essential medium (MEM, Thermo Fisher Scientific, Waltham, MA, USA) supplemented with 10% fetal bovine serum (FBS, Thermo Fisher Scientific), non-essential amino acids (Thermo Fisher Scientific), 100 U/mL penicillin and 100 µg/mL streptomycin (Thermo Fisher Scientific). Confluent cells were harvested using 0.25% trypsin-EDTA (Thermo Fisher Scientific) for 2–3 min and subcultured at a 1:2 to 1:4 split ratio. Cells were passed every 3–4 days and maintained for no more than 20 passages. To induce cellular quiescence, CCD-34Lu cells were grown to confluence and maintained under non-proliferative conditions. Growth medium was refreshed every 2–3 days. At confluence, cells were serum-starved by incubation in MEM supplemented with 0.1% FBS, non-essential amino acids, and 1% penicillin/streptomycin for 5 to 30 days, depending on the specific experimental requirements.

### siRNA transfections and proliferation assays

Transient silencing of DCTPP1 expression was achieved using siRNA oligonucleotide pools (ON-TARGET plus smart pool, Dharmacon, Perkin-Elmer Life Sciences, Waltham, MA, USA) specific for DCTPP1. Subconfluent cells [12, 13] were transfected using an siRNA oligonucleotide pool comprising four specific sequences targeting the following regions of DCTPP1 mRNA: 5’-GGCGAUAACUUCUAGAUUA-3’; 5’-CCCAGUAGGAUGUCAUGUA-3’; 5’-UCUUAGAGAUUGAAGGCUG-3’ and 5’-CCGCAAGUAUACAGAGAAUUG-3’. A non-targeting siRNA was used as control (ON-TARGET plus non-targeting pool, Dharmacon).

For cycling cells, siRNA transfections were performed overnight using the jetPRIME® transfection reagent (Polypus, Illkirch, France), followed by the addition of fresh medium. Usually, 66 or 350 pmol of siRNA was used per well in a 6-well plate or 100-mm cell culture dish, respectively. In non-proliferative conditions, the lipofectamine RNAiMAX reagent (Thermo Fisher Scientific) was used for transfection. Briefly, cells were seeded in 60 mm dishes and maintained until full confluence (100%) was reached. At this point, cells were switched to serum-free medium and transfected with 55 pmol of an siRNA pool in serum-free DMEM (0.1% FBS, no antibiotics). Four days post-transfection, the medium was refreshed by replacing half with fresh starvation medium. After 2 additional days, cells were subjected to a second round of siRNA transfection (55 pmol) in DMEM containing 0.1% FBS. Samples were collected 3 days after the second transfection, ensuring a total incubation period of 10 days with siRNAs, without replating. For extended silencing under quiescent conditions, the protocol was repeated as necessary to achieve final silencing durations of 20 or 30 days.

For proliferation assays, cycling cells were seeded into 96-well plates (5 ×10^3^ cells/well) 24 h after transfection. At 24 h intervals, cells were incubated with 20 µL of resazurin (1.1 mg/mL, Merck/Sigma-Aldrich, Darmstadt, Germany) for 2 h at 37 °C in the dark. Cell growth was quantified by measuring fluorescence at an excitation wavelength of 570 nm and an emission wavelength of 590 nm using a Tecan Infinite 200 microplate reader. To assess cell viability under non-proliferative conditions a crystal violet assay was employed, as previously described [39]. Briefly, cells were seeded in 12-well plates and grown to confluence and the FBS concentration was then reduced to 0.1%. After 5 or 10 days of serum starvation, cells were gently washed twice with water, and residual liquid was removed by gently inverting the plate. Subsequently, 500 µL of 0.5% crystal violet staining solution was added per well and plates were incubated for 20 minutes at room temperature on a bench rocker at 20 oscillations per minute. The plates were washed four times with water and air-dried overnight. To quantify the staining, crystal violet was solubilized by adding 500 µL of methanol per well, and plates were incubated for 20 min at room temperature with shaking, protected from light. Absorbance was measured at 570 nm using a microplate reader (Tecan Infinite 200).

### Cells extracts and western blotting

Cells were washed once with ice-cold PBS and subsequently resuspended in 1 mL of RIPA lysis buffer per well in a 6-well plate (25 mM Tris·HCl, pH 7.6, 150 mM NaCl, 1% NP-40, 1% sodium deoxycholate, 0.1% SDS; Merk/Sigma-Aldrich), supplemented with 1X Halt Protease/phosphatase inhibitors (Thermo Fisher Scientific). Cell lysates were incubated on ice for 10 min. Following incubation, the lysates were centrifuged at 14,000 g for 15 min at 4 °C, and the resulting supernatants were stored at −20 °C. Protein concentrations were determined using the Bradford assay (Bio-Rad, Hercules, CA, USA). For western blot analysis, protein lysates were boiled for 5 min and samples (equal amounts of protein, 10 µg) were resolved by SDS-PAGE and transferred onto polyvinylidene difluoride (PVDF) membranes (Bio-Rad). Membranes were incubated overnight at 4 °C with the following primary antibodies: rabbit polyclonal anti-DCTPP1 and anti-dUTPase (1:5000 and 1:10000, respectively, both generated in our laboratory) [12], mouse monoclonal anti-TK1 (1:1000, Santa Cruz Biotechnology Dallas, Texas, USA; sc-377211), mouse monoclonal anti-dCK (1:3000, Santa Cruz Biotechnology; sc-393099), mouse monoclonal anti-DCTD (1:1000, Santa Cruz Biotechnology; sc-376659), rabbit polyclonal anti-TS (1:1000, Santa Cruz Biotechnology; sc-134525), rabbit polyclonal anti-p53R2 (1:3000, Merk/Sigma-Aldrich; PRS2383), rabbit polyclonal anti-SAMHD1 (1:1000, Antibodies Online, Limerick PA, USA; ABIN3187981), rabbit polyclonal anti-TK2 (1:1000, Acris, OriGene Technologies, Inc., Rockville, MD,USA; AP13622PU-N), and mouse monoclonal anti-tubulin (1:10,000, Merk/Sigma-Aldrich; T5168).

After incubation, membranes were washed (PBS-Tween 0.3%) and incubated with the appropriate secondary antibodies (anti-rabbit HRP-conjugated antibody, 1:5000; W4011 or anti-mouse HRP-conjugated antibody, 1:3000; W4021, Promega, Madison, WI, USA) for 60 min at room temperature. Protein signals were detected using ECL reagents (GE Healthcare, Chicago, IL, USA). Multiple exposures were obtained to ensure that densitometric analysis was performed within the linear range of the signal. The films were scanned, and densitometric analysis was conducted using NIH ImageJ software (National Institutes of Health, Bethesda, MD, USA).

### Immunocytochemistry and quantitative image analysis

For immunocytochemistry, transfected cells were plated at a density of 25,000 cells per well on sterile coverslips in a 12-well plate and processed at the indicated time post-transfection. Under non-proliferative conditions, cells were harvested using Accutase Cell Dissociation Reagent (Gibco, Grand Island, NY, USA) instead of the 0.25% trypsin-EDTA used for proliferating cells. To label mitochondria, live cells were incubated with 250 nM MitoTracker™ Red CMXRos (Invitrogen, Carlsbad, CA, USA; M7512) for 30 min at 37 °C. Following labelling, cells were fixed and permeabilized as described previously [12]. Primary antibodies included a rabbit polyclonal anti-DCTPP1 and anti-dUTPase (1:2000), a mouse monoclonal anti-DCTD (1:100, Santa Cruz Biotechnology; sc-376659), a rabbit polyclonal anti-TS (1:1000, Santa Cruz Biotechnology; sc-134525), a mouse monoclonal anti-SAMHD1 (1:1000, Abcam Cambridge, UK; ab128107), a mouse monoclonal anti-TK1 (1:250; Santa Cruz Biotechnology; sc-377211), a mouse monoclonal anti-dCK (1:1000, Santa Cruz Biotechnology; sc-393099), a rabbit polyclonal anti-p53R2 (1:1000, Merk/Sigma-Aldrich; PRS2383), a rabbit polyclonal anti-TK2 (1:100, Acris; AP13622PU-N), and a mouse monoclonal anti-DNA (1:250, Merk/Sigma-Aldrich; CBL168). Secondary antibodies included goat anti-rabbit or goat anti-mouse Alexa Fluor® 488-conjugated antibodies (1:1000; Invitrogen, Thermo Fisher Scientific; A-11008 and A-11001, respectively). Cells were mounted using Vectashield mounting medium containing DAPI (Vector Laboratories Newark, CA, United States), and images were acquired using a Leica TCS-SP5 confocal microscope equipped with a 63 × 1.4 NA oil UV objective (HCX PLAPO CS) and a Leica TCS-SP8 confocal microscope with a 100×1.4 NA oil UV objective (HCX PLAPO CS). Images were collected using single excitation for each wavelength separately (488 nm Argon Laser line and a 500–545 nm emission band pass; HeNe Laser line and a 556-673 emission band pass; 405 nm UV diode and a 422–466 nm emission band pass). Thirty image sections of selected areas were acquired with a step size of 0.45 µm, and z-stack images analysed and processed using Leica Applied Systems (LAS AF6000) image acquisition software and NIH ImageJ software. The same laser intensity was maintained throughout each individual experiment to ensure consistency.

Colocalization measurements were performed on images where both fluorophores were properly aligned, ensuring no cross-talk and that the intensity remained within the microscope response range. Colocalization analysis was carried out using the Fiji colocalization plugin, and Mander’s correlation coefficient was determined. For each condition, an average of 30 independent cells from three independent experiments was analysed.

For DCTPP1 silencing experiments, cells were transiently transfected with siRNA oligonucleotide pools (ON-TARGETplus Smart Pool and ON-TARGETplus Non-targeting Pool, Dharmacon) using Jet Prime or RNAiMAX Lipofectamine, in proliferative or quiescent cells respectively. At 4 or 10 days post-transfection (in proliferative or quiescent cells respectively), cells were seeded onto sterile coverslips, fixed, and stained as described above.

For in situ mtDNA visualization experiments, images were acquired using confocal and super-resolution lightning imaging (with up to 120 nm lateral resolution) on a TCS-SP8 system. 3D reconstruction images were also obtained using super-resolution lightning imaging and high-resolution 3D confocal modules (LAS X 3.1.1. 15751).

### BrdU Incorporation and cell cycle analysis

Lung fibroblasts were seeded at a density of 9 × 10⁵ cells per 10 cm dish and transfected 24 h later with either the control or anti-DCTPP1 siRNAs. Four days post-transfection, cells were pulse-labeled with 10 µM BrdU (BD Biosciences, San Jose, CA, USA; 550891) for 1 h at 37 °C. After labeling, cells were trypsinized using 0.25% trypsin-EDTA and washed with 1X PBS. Cells were then fixed and permeabilized with 70% ice-cold ethanol overnight. Following fixation, cells were washed with washing solution (1X PBS and 0.2% Tween 20), incubated for 20 min with 4 M HCl and 1% Triton X -100, and washed three times with washing solution. Cells were then incubated overnight at 4 °C with anti-BrdU-FITC antibody (BD Biosciences; 556028) and diluted 1:5 in blocking solution. DNA was stained by incubating cells with 0.05 mg/mL propidium iodide and 0.05 mg/mL RNase at room temperature for 20 min. Cell suspensions were analysed using a FACSAria III cell sorter flow cytometer (BD Biosciences). Flow cytometry data were processed and FlowJo 7.6 software (Ashland, OR, USA) was used for cell cycle analysis.

### dNTP pools quantification

dNTPs pool determination was performed using the DNA polymerase assay [13], based on the incorporation of [^3^H]-dATP (Hartman Analytic, Braunschweig, Deutschland; 16.8 Ci/mmol) or [^3^H]-dTTP (Perkin-Elmer Life Sciences; 20.7 Ci/mmol) into sequence-specific oligonucleotides by a DNA polymerase (*E.coli* Klenow fragment, New England Biolabs Ipswich, MA,USA). The oligonucleotides used in the assay were designed so that the incorporation of labeled dATP or dTTP is proportional to the amount of the respective dNTP present in the sample [12]. Briefly, 2.2 × 10^6^ cells were extracted with 1.2 mL of 1:1 (v:v) methanol/water at −20 °C and the suspension was vortex-mixed. Samples underwent two freeze-thaw cycles (10 min each at dry ice/ethanol), followed by boiling for 5 min and centrifugation at 16,000 g for 20 min at 4 °C. The supernatants were collected, divided into 250 µL aliquots, and stored at −80 °C until further analysis. To determine dUTP and dTTP, samples were dried under vacuum (SpeedVac DNA 120 (Savant)) and then dissolved in 40 µL of buffer (34 mMTris/HCl, pH 7.8, and 5 mM MgCl_2_) with or without 30 ng of dUTPase and incubated 20 min at 37 °C. The reaction was stopped with 100% of ice-cold methanol and centrifuged at 16,000 g for 20 min at 4 °C, dried and used for the quantification of the dNTP pool. For dNTP determination, the dried samples were resuspended in 100 µL of reaction buffer containing 32 nM oligonucleotide primer, NE Buffer 2, 0.3 units of Klenow fragment of DNA polymerase I, and 0.025 nmol of [^3^H]-dATP or [^3^H]-dTTP. The reaction was incubated for 15 min at 25 °C and stopped by the addition of 10 mM EDTA, followed by heating at 75 °C for 20 min. DNA was precipitated with 10% (v/v) TCA for 30 min at 4 °C. The samples were filtered through Whatman GF/C glass microfiber filters (Cytiva life sciences, Marlborough, MA, USA), washed with 30 mL of 5% TCA, and dried with 3 mL of ethanol. Radioactivity was quantified using a LS 6500 Multi-Purpose scintillation counter (Beckman Coulter, Indianapolis, Indiana, USA). A standard curve for each dNTP was used for nucleotide quantification.

### Mitochondrial staining and flow cytometry

CCD-34Lu cells were seeded and transiently transfected with either siRNA control or siDCTPP1 for 4 or 10 days, in proliferating or quiescent states, respectively. Subsequently, 1 × 10⁶ cells were incubated with 5 mL of complete medium containing 50 nM MitoTracker™ Red CMXRos (Thermo Fisher Scientific; M7512) and 100 nM MitoTracker™ Green FM (Thermo Fisher Scientific; M7514) for 20 min at 37 °C, protected from light. To prevent spectral overlap between the MitoTracker™ Red and MitoTracker™ Green channels, fluorescence minus one (FMO) controls were included, which contained all fluorochromes except the dye of interest. Samples were acquired by flow cytometry, unfixed, within 2 h of staining completion. A minimum of 10,000 events were collected for analysis. Flow cytometry data were acquired using a FACSAria III cell sorter (BD Biosciences) and analysed using FlowJo software (version 7.6). Compensation for fluorescence parameters was performed with FlowJo using appropriate compensation control. Flow cytometry data were reported as the geometric mean fluorescence intensity (MFI) in arbitrary units, with the fluorescence values expressed on a log10 scale. Mitochondrial membrane potential was quantified by calculating the ratio of the MFI of MitoTracker™ Red CMXRos, which is sensitive to changes in membrane potential, to the MFI of MitoTracker™ Green FM, which correlates with mitochondrial mass and remains unaffected by membrane potential. The resulting ratio was used to determine the normalized mitochondrial membrane potential, expressed as a percentage.

### PCR based determination of mitochondrial DNA copy number

Mitochondrial and nuclear DNA were isolated from approximately 1 × 10⁶ cells using the DNeasy Blood and Tissue Kit (Qiagen, Steinhausen, Switzerland) according to the manufacturer’s protocol. DNA concentration and purity were assessed using a NanoDrop spectrophotometer. The relative mitochondrial DNA (mtDNA) content was determined by real-time quantitative PCR as previously described [19, 20]. Reactions were performed in triplicate in 96-well PCR plates (Thermo Fisher Scientific) with a final reaction volume of 25 µL per well, containing 20 ng of DNA, 5 µL of Power SYBR™ Green PCR Master Mix (Applied Biosystems, South San Francisco, CA 94080, USA), and 0.5 µM of each forward and reverse primer. PCR amplification was conducted using a CFX96 Touch Real-Time PCR Detection System. Two pairs of primers were used to amplify mtDNA and nuclear DNA reference regions: Cox2 and App1 [20] or tRNA-Leu (UUR) and B2-microglobulin [40], respectively. The primer sequences were as follows: Cox2: Mm-COXII-F: GAGCAGTCCCCTCCCTAGGA, Mm-COXII-R: GGTTTGATGTTACTGTTGCTTGATTT; App1: Mm -APP1 -F: CGGAAACGACGCTCTCATG, Mm-APP1-R: CCAGGCTGAATTCCCCAT; tRNA-Leu(UUR): Mm-tRNA-Leu(UUR)-F: CACCCAAGAAACAGGGTTTGT, Mm-tRNA-Leu(UUR)-R: TGGCCATGGGTATGTTGTTA; B2-microglobulin: Mm-B2-microglobulin-F:TGCTGTCCTCATGTTTGATGTATCT, Mm-B2-microglobulin-R:TCTCTGCTCCCAAACCTCTAAGT. The relative amount of mtDNA was calculated using the 2^−ΔΔCt^ method [41] and expressed as fold changes relative to the indicated control. The results are represented as the mtDNA/nDNA ratio, normalized to the control sample and expressed as a percentage of relative mtDNA content.

### Citrate synthase activity assay

Citrate synthase activity was measured in fresh cell extracts prepared from approximately 1 × 10⁶ cells/mL, in triplicate, following the manufacturer’s instructions for the Citrate Synthase Assay Kit (Merk/Sigma-Aldrich). The specific activity of citrate synthase was determined using a UV-Vis spectrophotometer (Cary 100-Bio, Varian, Temecula, CA, USA), employing a kinetic program to monitor absorbance changes. The baseline absorbance corresponding to endogenous thiol or deacetylase activity was recorded and subsequently subtracted from the total absorbance following the addition of oxaloacetate. Citrate synthase activity was quantified by measuring the change in absorbance over a 1.5-minute period. The final enzyme activity was expressed as nmol of product formed per minute per milligram of protein (nmol/min × mg protein).

### Statistical data and reproducibility

Data were analysed using GraphPad Prism version 10 (GraphPad, Inc., La Jolla, CA, USA) and are presented as individual data points and/or mean ± standard deviation (S.D). The specific statistical tests applied in each case are described in the figure legends, along with the number of replicates for each experiment. Statistical significance was defined according to APA format as follows: n.s. *p* < 0.12, *p < 0.33, **p < 0.02, and ***p < 0.001. Representative images of immunofluorescence and western blotting are shown from three independent experiments. Original western blot data are provided in the supplementary figures.

## Supplementary information

Supplementary Figure 1

Supplementary Figure 2

Supplementary Figure 3

Supplementary Figure 4

Supplementary Figure 5

Supplementary legends_FINAL

Supplementary Original western blot

## Data Availability

All relevant data supporting the key findings of this study are available within the article and its Supplementary Information files or from corresponding authors on request.
