# Supplementary material for: DCTPP1 orchestrates dCTP pool dynamics and mtDNA stability in quiescent cells
Source: Cell Death Dis. 2026 Mar 26;17(1):404. doi: 10.1038/s41419-026-08632-1 (PMC13139568; doi:10.1038/s41419-026-08632-1)
Supplement: Supplementary file 6 — Supplementary legends_FINAL [file 41419_2026_8632_MOESM6_ESM.docx]

**SUPPLEMENTARY FIGURES LEGENDS**

**Supplementary Fig 1**. **Analysis of subcellular localization of pyrimidine metabolism enzymes in a CCD-34 Lu quiescent cell model.**

**A** Immunofluorescence (IF) staining of selected pyrimidine metabolism enzymes in CCD-34Lu cells. Left panel: representative images of cycling cells. Right panel: quiescent cells maintained under serum starvation for 10 days following contact inhibition. Mitochondria are labeled with MitoTracker™ Red (red channel), nuclei are stained with DAPI (blue channel), and the enzyme of interest is shown in green. Scale bar, 10 µm. **B** Quantification of total fluorescence intensity signal, expressed as CTCF (corrected total cell fluorescence), was performed using Fiji software from approximately 30 cells per condition. Values are presented as mean ± S.D. (n ≥ 30). Statistical analysis was performed using an unpaired t-test.

**Supplementary Fig 2. Immunofluorescence characterization of pyrimidine metabolism enzymes upon DCTPP1 knockdown in CCD-34 Lu cells.**

**A** Immunofluorescence staining of pyrimidine metabolism enzymes in cycling CCD-34 Lu cells transfected with siCtrl (left panel) or siDCTPP1 (right panel) after 4 days of silencing. Mitochondria are labeled with MitoTracker™ Red (red channel), nuclei are stained with DAPI (blue channel), and the enzyme of interest is shown in green. Bar, 10 µm. **B** Quantification of total fluorescence intensity signal, expressed as corrected total cell fluorescence (CTCF), was performed using Fiji software, based on the analysis of approximately 30 cells per experiment. All values are shown as mean ± SD (n ≥ 30). Statistical significance was assessed using an unpaired two-sided Student’s t-test.

**Supplementary Fig 3. Immunofluorescence panel of pyrimidine metabolism enzymes upon DCTPP1 knockdown in resting CCD-34Lu cells**

**A** Representative immunofluorescence images of CCD-34Lu quiescent cells transfected with either siCtrl (left panel) or siDCTPP1 (right panel) for 10 days. Mitochondria are labeled with MitoTracker™ Red (red channel), nuclei are stained with DAPI (blue channel), and the enzyme of interest is shown in green. Scale bar, 10 µm. Bar, 10 µm. **B** Fluorescence intensity, expressed as corrected total cell fluorescence (CTCF), was quantified using Fiji software from approximately 30 cells per condition. Results are shown as mean ± SD (n ≥ 30). Statistical significance was determined using an unpaired two-tailed Student’s t-test.

**Supplementary Fig 4. *In situ* mtDNA staining by immunofluorescence.**

**A** Representative immunofluorescence images of mtDNA-positive dots in proliferative and quiescent CCD-34Lu cells are shown; red: mitochondria marker (MitoTracker™ Red), green: DCTPP1, blue: nuclei (DAPI), and grey: mtDNA detected using anti-DNA antibody (clone AC-30-10, CBL186). Scale bar; 10 µm. **B** Quantification of mtDNA-positive dots was performed using Fiji software on 30 individual cells per condition (n ≥ 30). Statistical analysis was performed using an unpaired two-tailed Student's t-test for panels.

**Supplementary Fig 5. Mitochondrial function evaluation by citrate synthase activity.**

Citrate synthase activity was measured from fresh cell extracts (1-10^6^ cells/ml) in triplicate. Specific activity was measured using a UV-Vis spectrophotometer on a kinetic program. The baseline of endogenous thiol or deacetylase activity was recorded and subtracted from the total activity recorded from the absorbance changes for 1.5 minutes after the addition of oxaloacetate. CS activity was expressed as µmol x min^-1^ x mg^-1^ protein. **A** proliferative CCD-34Lu cells versus quiescent cells. **B** Proliferative CCD ‑34Lu cells upon DCTPP1 depletion by siRNA after 4-days of transfection. **C** Quiescent CCD-34Lu cells transfected with either siCtrl or siDCTPP1 for 10 days. BAY‑10, an inhibitor of mitochondrial complex I, also known as NADH:ubiquinone oxidoreductase, and an inducer of ferroptosis, was used as mitochondrial dysfunction
