# Supplementary material for: DCTPP1 orchestrates dCTP pool dynamics and mtDNA stability in quiescent cells
Source: Cell Death Dis. 2026 Mar 26;17(1):404. doi: 10.1038/s41419-026-08632-1 (PMC13139568; doi:10.1038/s41419-026-08632-1)
Supplement: Supplementary file 7 — Supplementary Original western blot [file 41419_2026_8632_MOESM7_ESM.pdf]

FIGURE 1

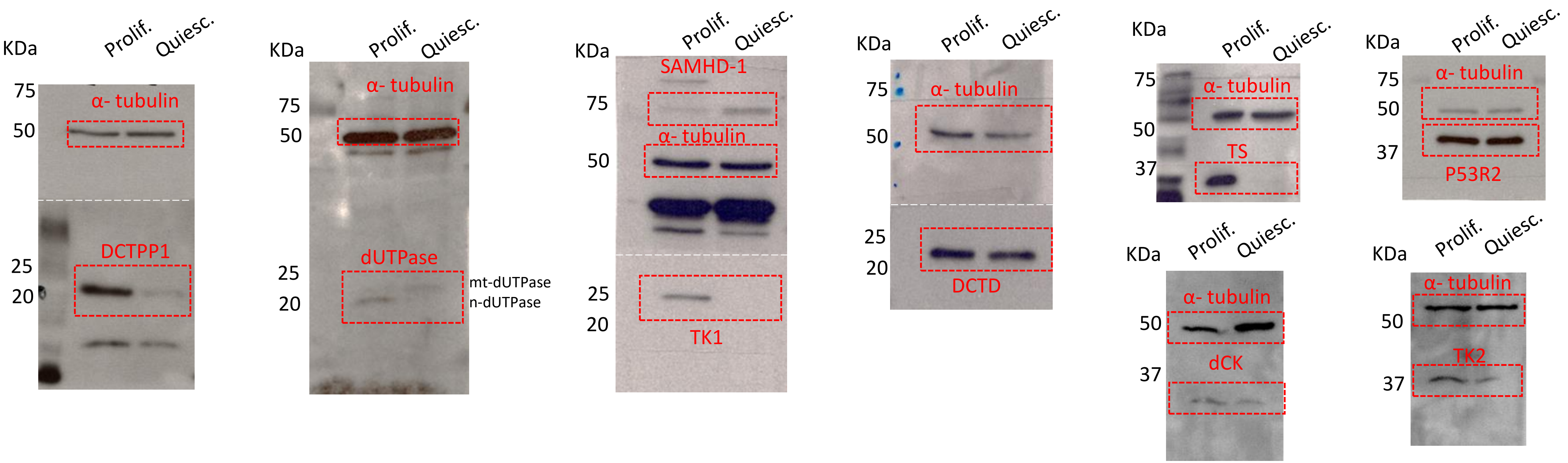

Figure 1. Original Western blots of pyrimidine metabolism enzymes in resting and proliferating cells. Full-length blots corresponding to Figure 1C are shown, with regions used in the main figure outlined in red. Tubulin was used as a loading control. PVDF membranes were occasionally cut post-transfer to allow parallel antibody incubations; cuts are marked by white dotted lines. "Prolif." denotes proliferating cells, and "Quiesc." refers to cells after a 10-day quiescence period.

FIGURE 2

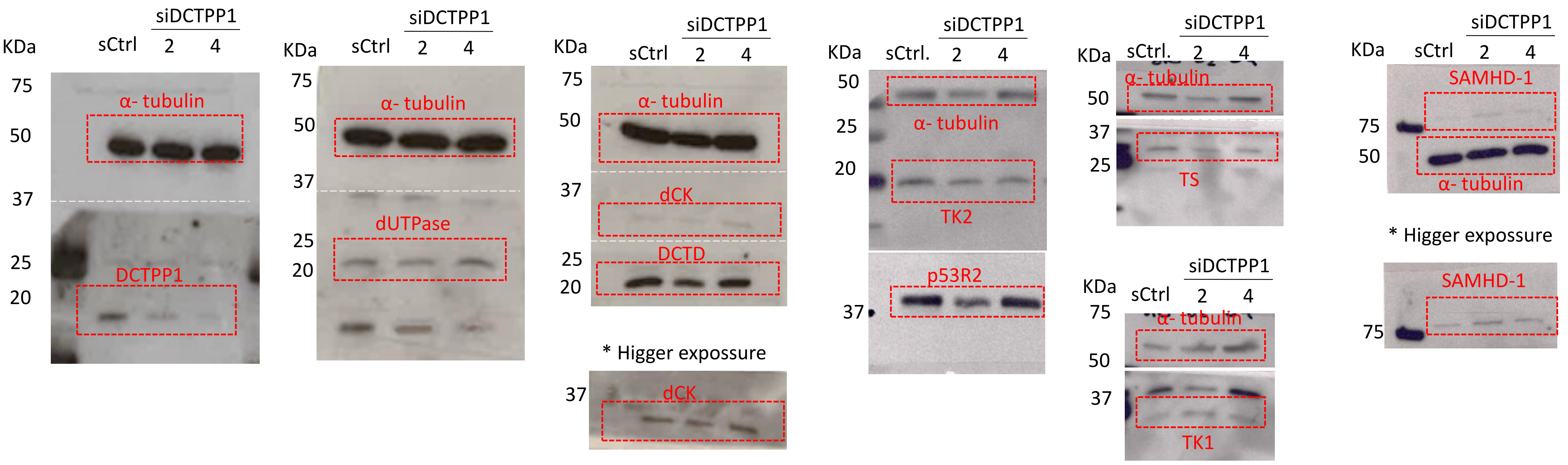

Figure 2. Original Western blots of pyrimidine metabolism enzymes following DCTPP1 depletion in proliferating cells. Western blot analysis was performed on proliferating CCD-34Lu cells transfected with siCtrl or siDCTPP1 for 2 and 4 days. Full-length blots corresponding to Figure 2C are shown, with regions used in the main figure outlined in red. Tubulin was used as a loading control, with non-saturated exposures to ensure accurate quantification. In some cases, PVDF membranes were cut post-transfer to allow parallel antibody incubations; these cuts are indicated by white dotted lines. Higher-exposure images for dCK and SAMHD1 are marked with an asterisk (*).

FIGURE 3

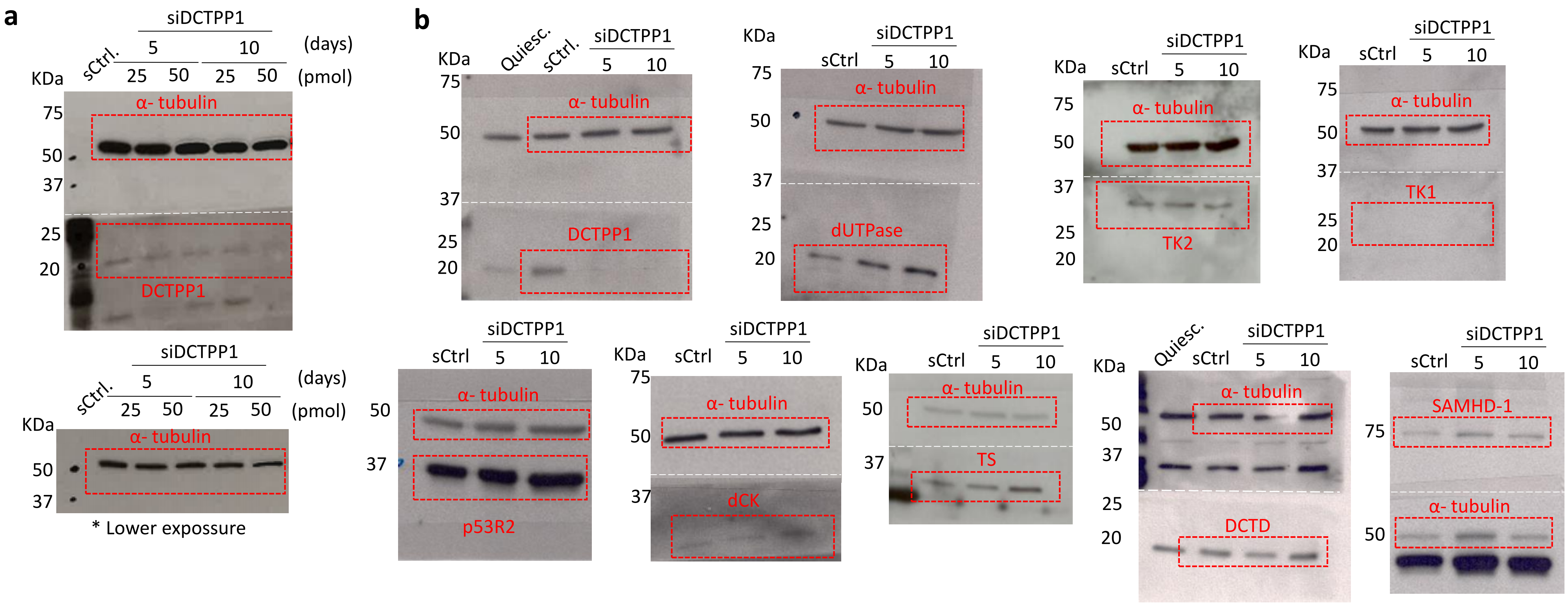

Figure 3. Original Western blots showing the effect of DCTPP1 depletion in quiescent CCD-34Lu cells. **a** Western blot analysis of DCTPP1 levels in quiescent CCD-34Lu cells transfected with siCtrl or siDCTPP1 (25 or 50 pmol) for 5 and 10 days (corresponding to Fig. 3A). **b** Full-length blots corresponding to Figure 3C, with regions used in the main figure outlined in red. Tubulin was used as a loading control. PVDF membranes were sometimes cut post-transfer to allow parallel antibody incubations; cuts are indicated by white dotted lines. Lower-exposure for TUB (a) marked with an asterisk (*).

FIGURE 6

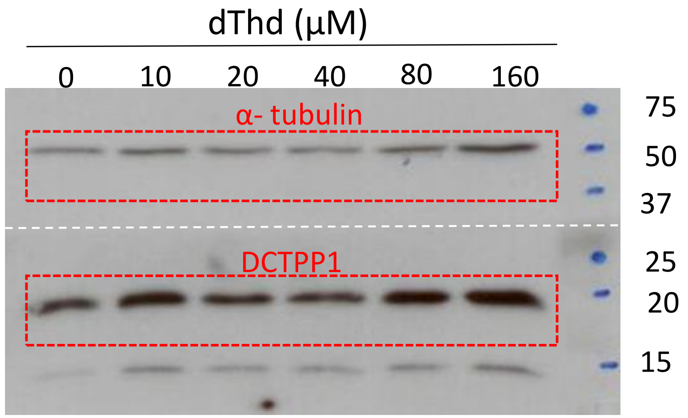

Figure 6. Original blot of DCTPP1 expression in the presence of dThd overload.
Full-size images of the Western blots shown in Fig. 6A are presented here, with the regions outlined in the main figure delineated by a red dashed line.
